# *g*-Weak Contraction in Ordered Cone Rectangular Metric Spaces

**DOI:** 10.1155/2013/810732

**Published:** 2013-05-27

**Authors:** S. K. Malhotra, J. B. Sharma, Satish Shukla

**Affiliations:** ^1^Department of Mathematics, Govt. S.G.S.P.G. College Ganj Basoda, Vidisha 464221, India; ^2^Department of Mathematics, Choithram College of Professional Studies, Dhar Road, Indore 453001, India; ^3^Department of Applied Mathematics, Shri Vaishnav Institute of Technology & Science, Gram Baroli Sanwer Road, Indore 453331, India

## Abstract

We prove some common fixed-point theorems for the ordered *g*-weak contractions in cone rectangular metric spaces without assuming the normality of cone. Our results generalize some recent results from cone metric and cone rectangular metric spaces into ordered cone rectangular metric spaces. Examples are provided which illustrate the results.

## 1. Introduction and Preliminaries

 There are a number of generalizations of metric spaces. One such generalization is obtained by replacing the real valued metric function with a vector valued metric function. In the mid-20th century (see [1]), the notions of K-metric and K-normed spaces were introduced, in such spaces an ordered Banach space instead of the real numbers was used as a codomain for metric function. Indeed, this idea of replacement of real numbers by an ordered “set” can be seen in [2, 3] (see also references therein). Huang and Zhang [4] reintroduced such spaces under the name of cone metric spaces, defining convergent and Cauchy sequence in terms of interior points of underlying cone. They proved the basic version of the fixed-point theorem with the assumption that the cone is normal. Subsequently several authors (see, e.g., [5–14]) generalized the results of Huang and Zhang. In [13], Rezapour and Hamlbarani removed the normality of cone and proved the results of Huang and Zhang in nonnormal cone metric spaces. 

In [15], Branciari introduced a class of generalized metric spaces with replacing triangular inequality by similar ones which involve four or more points instead of three and improved Banach contraction principle. Azam and Arshad [16] proved fixed-point result for Kannan-type contraction in rectangular metric spaces. After the work of Huang and Zhang [4], Azam et al. [17] introduced the notion of cone rectangular metric spaces and proved fixed-point result for Banach-type contraction in cone rectangular space. Samet and Vetro [18] obtained the fixed-point results in c-chainable cone rectangular metric spaces.

Ordered normed spaces and cones have applications in applied mathematics, for instance, in using Newton's approximation method [19] and in optimization theory [20]. The existence of fixed point in partially ordered sets was investigated by Ran and Reurings [21] and then by Nieto and Rodríguez-López [22]. Fixed-point results in ordered cone metric spaces were obtained by several authors (see, e.g., [11, 23–25]). Very recently, Malhotra et al. [26] proved the fixed-point results in ordered cone rectangular metric spaces for Reich-type contractions.

The notion of *g*-weak contraction is introduced by Vetro (see [14]) in cone metric spaces. In this paper, we prove some common fixed point theorems for *g*-weak contractions in ordered cone rectangular metric spaces. Our results generalize and extend the results of Huang and Zhang [4], Azam et al. [17], Azam and Arshad [16], Malhotra et al. [26], and the result of Vetro [14] on ordered cone rectangular metric spaces.

We need the following definitions and results, consistent with [4, 20].

**Definition 1 (see [4])**

Let *E* be a real Banach space and *P* a subset of *E*. The set *P* is called a cone if

- *P*  is closed, nonempty, and *P* ≠ {*θ*}; here *θ* is the zero vector of *E*;
- *a*, *b* ∈ ℝ, *a*, *b* ≥ 0, *x*, *y* ∈ *P*⇒*ax* + *by* ∈ *P*;
- *x* ∈ *P* and −*x* ∈ *P*⇒*x* = *θ*.

Given a cone *P* ⊂ *E*, we define a partial ordering “⪯” with respect to *P* by *x*⪯*y* if and only if *y* − *x* ∈ *P*. We write *x*≺*y* to indicate that *x*⪯*y* but *x* ≠ *y*, while *x* ≪ *y* if and only if *y* − *x* ∈ *P* ^0^, where *P* ^0^ denotes the interior of *P*.

Let *P* be a cone in a real Banach space *E*; then *P* is called normal, if there exists a constant *K* > 0 such that for all *x*, *y* ∈ *E*,

(1) $$\begin{array}{c} \theta ⪯x⪯y\;\;\text{implies}\;\;||x||\leq K||y||. \end{array}$$

The least positive number *K* satisfying the above inequality is called the normal constant of *P*.

**Definition 2 (see [4])**

Let *X* be a nonempty set and *E* a real Banach space. Suppose that the mapping *d* : *X* × *X* → *E* satisfies

- *θ*⪯*d*(*x*, *y*) for all *x*, *y* ∈ *X* and *d*(*x*, *y*) = *θ* if and only if *x* = *y*;
- *d*(*x*, *y*) = *d*(*y*, *x*) for all *x*, *y* ∈ *X*;
- *d*(*x*, *y*)⪯*d*(*x*, *z*) + *d*(*y*, *z*) for all *x*, *y*, *z* ∈ *X*.

Then, *d* is called a cone metric on *X*, and (*X*, *d*) is called a cone metric space. In the following, we always suppose that *E* is a real Banach space, and *P* is a solid cone in *E*; that is, *P* ^0^ ≠ *ϕ* and “⪯” is partial ordering with respect to *P*.

The concept of cone metric space is more general than that of a metric space because each metric space is a cone metric space with *E* = ℝ and *P* = [0, +*∞*).

For examples and basic properties of normal and nonnormal cones and cone metric spaces, we refer to [4, 13].

The following remark will be useful in sequel.

**Remark 3 (see [27])**

Let *P* be a cone in a real Banach space *E*, and *a*,  *b*,  *c* ∈ *P* we then have the following

- If *a*⪯*b* and *b* ≪ *c* then *a* ≪ *c*.
- If *a* ≪ *b* and *b* ≪ *c* then *a* ≪ *c*.
- If *θ*⪯*u* ≪ *c* for each *c* ∈ *P* ^0^, then *u* = *θ*.
- If *c* ∈ *P* ^0^ and *a*_*n*_ → *θ*, then there exist *n*_0_ ∈ *ℕ* such that, for all *n* > *n*_0_, we have *a*_*n*_ ≪ *c*.
- If *θ*⪯*a*_*n*_⪯*b*_*n*_ for each *n* and *a*_*n*_ → *a*,  *b*_*n*_ → *b*, then *a*⪯*b*.
- If *a*⪯*λa*, where 0 ≤ *λ* < 1, then *a* = *θ*.

**Definition 4 (see [17])**

Let *X* be a nonempty set. Suppose that the mapping *d* : *X* × *X* → *E* satisfies

- *θ*⪯*d*(*x*, *y*) for all *x*, *y* ∈ *X* and *d*(*x*, *y*) = *θ* if and only if *x* = *y*;
- *d*(*x*, *y*) = *d*(*y*, *x*) for all *x*, *y* ∈ *X*;
- *d*(*x*, *y*)⪯*d*(*x*, *w*) + *d*(*w*, *z*) + *d*(*z*, *y*) for all *x*, *y* ∈ *X* and for all distinct points *w*, *z* ∈ *X* − {*x*, *y*} (rectangular property).

Then, *d* is called a cone rectangular metric on *X*, and (*X*, *d*) is called a cone rectangular metric space. Let {*x*_*n*_} be a sequence in (*X*, *d*) and *x* ∈ *X*. If for every *c* ∈ *E*, with *θ* ≪ *c* there is *n*_0_ ∈ *ℕ* such that for all *n* > *n*_0_, *d*(*x*_*n*_, *x*) ≪ *c*, then {*x*_*n*_} is said to be convergent, {*x*_*n*_} converges to *x*, and *x* is the limit of {*x*_*n*_}. We denote this by lim⁡_*n*_ *x*_*n*_ = *x* or *x*_*n*_ → *x*, as *n* → *∞*. If for every *c* ∈ *E* with *θ* ≪ *c* there is *n*_0_ ∈ *ℕ* such that for all *n* > *n*_0_ and *m* ∈ *ℕ* we have *d*(*x*_*n*_, *x*_*n*+*m*_) ≪ *c*, then {*x*_*n*_} is called a Cauchy sequence in (*X*, *d*). If every Cauchy sequence is convergent in (*X*, *d*), then (*X*, *d*) is called a complete cone rectangular metric space. If the underlying cone is normal, then (*X*, *d*) is called normal cone rectangular metric space.

**Example 5**

Let *X* = *ℕ*, *E* = ℝ^2^, and *P* = {(*x*, *y*) : *x*, *y* ≥ 0}.

Define *d* : *X* × *X* → *E* as follows:

(2) $$\begin{array}{c} d\left(x,y\right)=\{\begin{array}{cc} \left(0,0\right) & \text{if}\;\;x=y,\\ \left(3,9\right) & \text{if}\;\;x\;\;\text{and}\;\;y\;\;\text{are}\;\;\text{in}\;\;\left\{1,2\right\},\;\;x\neq y,\\ \left(1,3\right) & \text{if}\;\;x\;\;\text{and}\;\;y\;\;\text{cannot}\;\;\text{both}\;\;\text{at}\\ & \;\text{a}\;\;\text{time}\;\;\text{in}\;\;\left\{1,2\right\},\;\;x\neq y. \end{array} \end{array}$$

Now (*X*, *d*) is a cone rectangular metric space but (*X*, *d*) is not a cone metric space because it lacks the triangular property:

(3) $$\begin{array}{c} \left(3,9\right)=d\left(1,2\right)>d\left(1,3\right)+d\left(3,2\right)=\left(1,3\right)+\left(1,3\right)=\left(2,6\right), \end{array}$$

as (3,9)−(2,6) = (1,3) ∈ *P*.

Note that in the above example (*X*, *d*) is a normal cone rectangular metric space. The following is an example of non-normal cone rectangular metric space.

**Example 6**

Let *X* = *ℕ*, *E* = *C*_ℝ_^1^[0,1] with ||*x*|| = ||*x*||_*∞*_ + ||*x*′||_*∞*_, and *P* = {*x* ∈ *E* : *x*(*t*) ≥ 0 for *t* ∈ [0,1]}. Then, this cone is not normal (see [13]).

Define *d* : *X* × *X* → *E* as follows:

(4) $$\begin{array}{c} d\left(x,y\right)=\{\begin{array}{cc} 0 & \text{if}\;\;x=y,\\ 3e^{t} & \text{if}\;\;x\;\;\text{and}\;\;y\;\;\text{are}\;\;\text{in}\;\;\left\{1,2\right\},\;\;\;x\neq y,\\ e^{t} & \text{if}\;\;x\;\;\text{and}\;\;y\;\;\text{cannot}\;\;\text{both}\;\;\text{at}\;\;\\ & \;\text{a}\;\;\text{time}\;\;\text{in}\;\;\left\{1,2\right\},\;\;\;x\neq y. \end{array} \end{array}$$

Then (*X*, *d*) is nonnormal cone rectangular metric space but (*X*, *d*) is not a cone metric space because it lacks the triangular property.

**Definition 7 (see [5])**

Let *f* and *g* be self-mappings of a nonempty set *X* and *C*(*f*, *g*) = {*x* ∈ *X* : *fx* = *gx*}. The pair (*f*, *g*) is called weakly compatible if *f* *gx* = *g* *fx* for all *x* ∈ *C*(*f*, *g*). If *w* = *fx* = *gx* for some *x* in *X*, then *x* is called a coincidence point of *f* and *g*, and *w* is called a point of coincidence of *f* and *g*.

**Definition 8**

If a nonempty set *X* is equipped with a partial order “⊑” and mapping *d* : *X* × *X* → *E* such that (*X*, *d*) is a cone rectangular metric space, then (*X*, ⊑, *d*) is called an ordered cone rectangular metric space. Let *f*, *g* : *X* → *X* be two mappings. The mapping *f* is called nondecreasing with respect to “⊑”, if for each *x*, *y* ∈ *X*, *x*⊑*y* implies *fx*⊑*fy*. The mapping *f* is called *g*-nondecreasing if for each *x*, *y* ∈ *X*, *gx*⊑*gy* implies *fx*⊑*fy*. A subset *𝒜* of *X* is called well ordered if for all the elements of *𝒜* are comparable; that is, for all *x*, *y* ∈ *𝒜* either *x*⊑*y* or *y*⊑*x*. *𝒜* is called *g*-well ordered if all the elements of *𝒜* are *g*-comparable; that is, for all *x*, *y* ∈ *𝒜* either *gx*⊑*gy* or *gy*⊑*gx*.

In the trivial case, that is, for *g* = *I*_*X*_ (the identity mapping of *X*), the *g*-well orderedness reduces into well orderedness. But, for nontrivial cases, that is, when *g* ≠ *I*_*X*_ the concepts of *g*-well orderedness and well orderedness are independent.

**Example 9**

Let *X* = {0,1, 2,3, 4}, let “⊑” be a partial order relation on *X* defined by ⊑ = {(0,0), (1,1),(2,2), (3,3), (4,4), (1,2), (2,3), (1,3), (1,4)}. Let *𝒜* = {0,1, 3}, *ℬ* = {1,4} and *g* : *X* → *X* be defined by *g*0 = 1,  *g*1 = 2,  *g*2 = 3,  *g*3 = 3,  *g*4 = 0. Then it is clear that *𝒜* is not well ordered but it is *g*-well ordered, while *ℬ* is not *g*-well ordered but it is well ordered.

Let (*X*, ⊑, *d*) be an ordered cone rectangular metric space *f*, *g* : *X* → *X* two mappings. The mapping *f* is called ordered Reich-type contraction if for all *x*, *y* ∈ *X* with *x*⊑*y*,  *λ*,  *μ*,  *δ* ∈ [0,1) such that *λ* + *μ* + *δ* < 1 and

(5) $$\begin{array}{c} d\left(fx,fy\right)⪯\lambda d\left(x,y\right)+\mu d\left(x,fx\right)+\delta d\left(y,fy\right). \end{array}$$

If (5) is satisfied for all *x*, *y* ∈ *X*, then *f* is called Reich contraction.

The mapping *f* is called an ordered *g*-weak contraction if

(6) $$\begin{array}{c} d\left(fx,fy\right)⪯\lambda d\left(gx,gy\right)+\mu d\left(gx,fx\right)+\delta d\left(gy,fy\right) \end{array}$$

for all *x*, *y* ∈ *X* with *gx*⊑*gy*, where *λ*, *μ*, and *δ* are nonnegative constants such that *λ* + *μ* + *δ* < 1. If inequality (6) is satisfied for all *x*, *y* ∈ *X*, then *f* is called a *g*-weak contraction.

Note that for *g* = *I*_*X*_ (the identity mapping of *X*) the ordered *g*-weak contraction reduces into the ordered Reich contraction.

Now, we can state our main results. 

## 2. Main Results

**Theorem 10**

Let (*X*, ⊑, *d*) be an ordered cone rectangular metric space *f*, *g* : *X* → *X* two mappings such that *f*(*X*) ⊂ *g*(*X*) and *g*(*X*) is complete. Suppose that the following conditions are satisfied:

- *f* is an ordered *g*-weak contraction, that is, satisfies (6);
- *f* is *g*-nondecreasing;
- there exists *x*_0_ ∈ *X* such that *gx*_0_⊑*fx*_0_;
- if {*gx*_*n*_} were any nondecreasing sequence in *X* converging to some *gz*, then *gx*_*n*_⊑*gz* for all *n* and *gz*⊑*g* *gz*.

Then, *f* and *g* have a coincidence point. Furthermore, if *f* and *g* are weakly compatible then they have a common fixed point. In addition, the set of common fixed points of *f* and *g* is *g*-well ordered if and only if the common fixed point of *f* and *g* is unique.

**Proof**

Starting with given *x*_0_ ∈ *X*, we define a sequence {*y*_*n*_} as follows: let *fx*_0_ = *gx*_1_ = *y*_1_ (which is possible as *f*(*X*) ⊂ *g*(*X*)). As *gx*_0_⊑*fx*_0_, we have *gx*_0_⊑*gx*_1_, and as *f* is *g*-nondecreasing, we obtain *fx*_0_⊑*fx*_1_. Again, *f*(*X*) ⊂ *g*(*X*) therefore let *fx*_1_ = *gx*_2_ = *y*_2_. Since *gx*_1_⊑*gx*_2_ and *f* is *g*-nondecreasing, we obtain *gx*_1_⊑*gx*_2_. On repeating this process, we obtain

(7) fx0⊑fx1⊑⋯⊑fxn⊑fxn+1⊑⋯,gx1⊑gx2⊑⋯⊑gxn⊑gxn+1⊑⋯,∀n∈ℕ.fxn−1=gxn=yn

Thus, {*y*_*n*_} = {*gx*_*n*_} is a nondecreasing sequence with respect to ⊑.

We will show that *f* and *g* have a point of coincidence. If, *y*_*n*_ = *y*_*n*+1_ for any *n* ∈ *ℕ*, we have *y*_*n*_ = *gx*_*n*_ = *fx*_*n*_; therefore, *y*_*n*_ is a point of coincidence of *f* and *g* with coincidence point *x*_*n*_. Therefore, we assume that *y*_*n*_ ≠ *y*_*n*+1_ for all *n* ∈ *ℕ*.

As, *gx*_*n*_⊑*gx*_*n*+1_ for all *n* ∈ *ℕ*, it follows from (6) that

(8) d(yn,yn+1)=d(fxn−1,fxn)⪯λd(gxn−1,gxn)+μd(gxn−1,fxn−1) +δd(gxn,fxn)=λd(yn−1,yn)+μd(yn−1,yn)+δd(yn,yn+1)=(λ+μ)d(yn−1,yn)+δd(yn,yn+1).

For simplicity, set *d*_*n*_ = *d*(*y*_*n*_, *y*_*n*+1_) for all *n* ∈ *ℕ*; then it follows from above inequality that

(9) $$\begin{array}{c} d_{n}⪯\frac{\lambda +\mu }{1-\delta }d_{n-1}=\alpha d_{n-1},\;\forall n\in \mathbb{N}, \end{array}$$

where *α* = (*λ* + *μ*)/(1 − *δ*) < 1 (as *λ* + *μ* + *δ* < 1). By repeating this process, we obtain

(10) $$\begin{array}{c} d_{n}⪯\alpha^{n}d_{0},\;\forall n\in \mathbb{N}. \end{array}$$

If *y*_*n*_ = *y*_*n*+*p*_ for any *n* ∈ *ℕ* and positive integer *p* > 1, then as *y*_*n*_⊑*y*_*n*+2_, it follows from (6) that

(11) d(yn,yn+1)=d(yn+p,yn+1)=d(fxn+p−1,fxn)⪯λd(gxn+p−1,gxn)+μd(gxn+p−1,fxn+p−1) +δd(gxn,fxn)=λd(yn+p−1,yn)+μd(yn+p−1,yn+p) +δd(yn,yn+1)=λd(yn+p−1,yn+p)+μd(yn+p−1,yn+p) +δd(yn,yn+1);

that is,

(12) $$\begin{array}{c} d_{n}⪯\lambda d_{n+p-1}+\mu d_{n+p-1}+\delta d_{n},\\ d_{n}⪯\alpha d_{n+p-1}. \end{array}$$

Repeating this process *p* times, we obtain

(13) $$\begin{array}{c} d_{n}⪯\alpha^{p}d_{n}<d_{n}\;\left(\text{as}\;\;\alpha =\frac{\lambda +\mu }{1-\delta }<1\right), \end{array}$$

a contradiction. Therefore, we can assume that *y*_*n*_ ≠ *y*_*m*_ for all distinct *n*, *m* ∈ *ℕ*.

Again, as *y*_*n*_⊑*y*_*n*+2_, we obtain from (6) and (10) that

(14) d(yn,yn+2)=d(fxn−1,fxn+1)⪯λd(gxn−1,gxn+1)+μd(gxn−1,fxn−1) +δd(gxn+1,fxn+1)=λd(yn−1,yn+1)+μd(yn−1,yn) +δd(yn+1,yn+2)⪯λ[d(yn−1,yn)+d(yn,yn+2)+d(yn+2,yn+1)] +μd(yn−1,yn)+δd(yn+1,yn+2)=λ[dn−1+d(yn,yn+2)+dn+1] +μdn−1+δdn+1;

that is,

(15) d(yn,yn+2)⪯λ+μ1−λdn−1+λ+δ1−λdn+1⪯λ+μ1−λαn−1d0+λ+δ1−λαn+1d0⪯λ+μ+[λ+δ]α21−λαn−1d0.

As *α* < 1, we have

(16) $$\begin{array}{c} d\left(y_{n},y_{n+2}\right)⪯\frac{2\lambda +\mu +\delta }{1-\lambda }\alpha^{n-1}d_{0},\\ d\left(y_{n},y_{n+2}\right)⪯\beta \alpha^{n-1}d_{0},\;\forall n\in \mathbb{N}, \end{array}$$

where *β* = (2*λ* + *μ* + *δ*)/(1 − *λ*) ≥ 0.

For the sequence {*y*_*n*_}, we consider *d*(*y*_*n*_, *y*_*n*+*p*_) in two cases.

If *p* is odd say 2*m* + 1, then using rectangular inequality and (10), we obtain

(17) d(yn,yn+2m+1)⪯d(yn+2m,yn+2m+1)+d(yn+2m−1,yn+2m) +d(yn,yn+2m−1)=dn+2m+dn+2m−1+d(yn,yn+2m−1)⪯dn+2m+dn+2m−1+dn+2m−2+dn+2m−3 +⋯+dn⪯αn+2md0+αn+2m−1d0+αn+2m−2d0 +⋯+αnd0=[α2m+α2m−1+⋯+1]αnd0⪯αn1−αd0.

Therefore,

(18) $$\begin{array}{c} d\left(y_{n},y_{n+2m+1}\right)⪯\frac{\alpha^{n}}{1-\alpha }d_{0}. \end{array}$$

If *p* is even, say 2*m*, then using rectangular inequality and (10) and (16), we obtain

(19) d(yn,yn+2m)⪯d(yn+2m−1,yn+2m)+d(yn+2m−1,yn+2m−2) +d(yn+2m−2,yn)=dn+2m−1+dn+2m−2+d(yn+2m−2,yn)⪯dn+2m−1+dn+2m−2+dn+2m−3+dn+2m−4 +⋯+dn+2+d(yn,yn+2)⪯αn+2m−1d0+αn+2m−2d0+⋯+αn+2d0 +βαn−1d0=[α2m−1+α2m−2+⋯+α2]αnd0+βαn−1d0⪯αn1−αd0+βαn−1d0.

Therefore,

(20) $$\begin{array}{c} d\left(y_{n},y_{n+2m}\right)⪯\frac{\alpha^{n}}{1-\alpha }d_{0}+\beta \alpha^{n-1}d_{0}. \end{array}$$

As *β* ≥ 0 and 0 ≤ *α* < 1, we have (*α* ^*n*^/(1 − *α*))*d*_0_ → *θ* and *βα* ^*n*−1^ *d*_0_ → *θ*. So, it follows from (18), (20), and (a), (d) of Remark 3 that for every *c* ∈ *E* with *θ* ≪ *c* there exists *n*_0_ ∈ *ℕ* such that *d*(*y*_*n*_, *y*_*n*+2*m*+1_) ≪ *c* and *d*(*y*_*n*_, *y*_*n*+2*m*_) ≪ *c* for all *n* > *n*_0_. Thus, {*y*_*n*_} = {*gx*_*n*_} is a Cauchy sequence in *g*(*X*). As *g*(*X*) is complete, there exist *z*, *u* ∈ *X* such that

(21) $$\begin{array}{c} \underset{n\to \infty }{\lim}y_{n}=\underset{n\to \infty }{\lim}gx_{n}=\underset{n\to \infty }{\lim}fx_{n-1}=gz=u. \end{array}$$

We will show that *fz* = *gz* = *u*.

Now,

(22) d(u,fz)⪯d(u,yn)+d(yn,yn+1)+d(yn+1,fz)=d(u,yn)+dn+d(yn+1,fz).

From (iv), we have *gx*_*n*_⊑*gz*; that is,  *y*_*n*_⊑*u*; therefore it follows from (6) that

(23) d(yn+1,fz)=d(fxn,fz)⪯λd(gxn,gz)+μd(gxn,fxn)+δd(gz,fz)=λd(yn,u)+μd(yn,yn+1)+δd(u,fz)⪯λd(yn,u)+μdn +δ[d(u,yn)+d(yn,yn+1)+d(yn+1,fz)]=[λ+δ]d(yn,u)+[μ+δ]dn+δd(yn+1,fz);

that is,

(24) $$\begin{array}{c} d\left(y_{n+1},fz\right)⪯\frac{\lambda +\delta }{1-\delta }d\left(y_{n},u\right)+\frac{\mu +\delta }{1-\delta }d_{n}. \end{array}$$

In view of (10), (21), and (a), (d) of Remark 3, for every *c* ∈ *E* with *θ* ≪ *c*, there exists *n*_1_ ∈ *ℕ* such that *d*(*y*_*n*_, *u*) ≪ *c*(1 − *δ*)/2(*λ* + *δ*), *d*_*n*_ ≪ *c*(1 − *δ*)/2(*μ* + *δ*) for all *n* > *n*_1_. Therefore, it follows from above inequality that

(25) $$\begin{array}{c} d\left(y_{n+1},fz\right)\ll c\;\forall n>n_{1}\;\;\text{and}\;\;\text{every}\;\;c\in E\;\;\text{with}\;\;\theta \ll c. \end{array}$$

Therefore, again with same arguments, from (22) and (c) of Remark 3, we obtain that *d*(*u*, *fz*) = *θ*; that is,  *fz* = *gz* = *u*. Thus, *z* is a coincidence point and *u* is point of coincidence of *f* and *g*.

Now suppose that *f* and *g* are weakly compatible; then we have *fu* = *f* *gz* = *g* *fz* = *gu*. As *gz*⊑*g* *gz*, therefore using (6) we obtain

(26) d(u,fu)=d(fz,ffz)⪯λd(gz,gfz)+μd(gz,fz)+δd(gfz,ffz)=λd(gz,fgz)+μd(gz,gz)+δd(fgz,fgz)=λd(u,fu).

As *λ* ∈ [0,1), it follows from (f) of Remark 3 and the above inequality that *d*(*u*, *fu*) = *θ*; that is, *fu* = *u* = *gu*. Thus, *u* is a common fixed point of *f* and *g*.

Suppose the set of common fixed points of *f* and *g*, that is, *ℱ*, is *g*-well ordered and *u*, *v* ∈ *ℱ*. As *ℱ* is *g*-well ordered, let, for example, *gu*⊑*gv*. Then, it follows from (6) that

(27) d(u,v)=d(fu,fv)⪯λd(gu,gv)+μd(gu,fu)+δd(gv,fv)=λd(u,v)+μd(u,u)+δd(v,v)=λd(u,v).

As *λ* ∈ [0,1), it follows from (f) of Remark 3 that *d*(*u*, *v*) = *θ*; that is,  *u* = *v*. Therefore, the common fixed point of *f* and *g* is unique. For converse, let common fixed point be unique, then *ℱ* will be singleton and therefore *g*-well ordered.

**Remark 11**

For the existence of common fixed point of *f* and *g*, Vetro [14] used the condition

(28) $$\begin{array}{c} fgx=ggx\;\text{whenever}\;\;fx=gx. \end{array}$$

Here, we have used the weak compatibility of mappings *f* and *g*, and it is obvious that the condition used by Vetro implies the weak compatibility of mappings *f* and *g*.

Taking *g* = *I*_*X*_ (identity mapping of *X*), we obtain the main result of [26].

**Corollary 12**

Let (*X*, ⊑, *d*) be an ordered complete cone rectangular metric space and *f* : *X* → *X* a mapping such that the following conditions are satisfied:

- *f* is an ordered Reich-type contraction, that is, satisfies (5);
- *f* is nondecreasing with respect to “⊑”;
- there exists *x*_0_ ∈ *X* such that *x*_0_⊑*fx*_0_;
- if {*x*_*n*_} is any nondecreasing sequence in *X* converging to some *z* then *x*_*n*_⊑*z*, for all *n*.

Then, *f* has a fixed point. In addition, the set of fixed points of *f* is well ordered if and only if the fixed point of *f* is unique.

With suitable values of control constants *λ*, *μ*, and *δ*, we obtain the following generalizations of Theorems 2.1 and 2.3 of Abbas and Jungck [5] on ordered cone rectangular metric spaces.

**Corollary 13**

Let (*X*, ⊑, *d*) be an ordered cone rectangular metric space *f*,  *g* : *X* → *X* two mappings such that *f*(*X*) ⊂ *g*(*X*) and *g*(*X*) is complete. Suppose that the following conditions are satisfied:

- *d*(*fx*, *fy*)⪯*λd*(*gx*, *gy*) for all *x*, *y* ∈ *X* with *gx*⊑*gy*, where *λ* ∈ [0,1);
- *f* is *g*-nondecreasing;
- there exists *x*_0_ ∈ *X* such that *gx*_0_⊑*fx*_0_;
- if {*gx*_*n*_} were any nondecreasing sequence in *X* converging to some *gz* then *gx*_*n*_⊑*gz*, for all *n* and *gz*⊑*g* *gz*.

Then, *f* and *g* have a coincidence point. Furthermore, if *f* and *g* are weakly compatible then they have a common fixed point. In addition, the set of common fixed points of *f* and *g* is *g*-well ordered if and only if the common fixed point of *f* and *g* is unique.

**Corollary 14**

Let (*X*, ⊑, *d*) be an ordered cone rectangular metric space and *f*, *g* :*X* → *X* two mappings such that *f*(*X*) ⊂ *g*(*X*) and *g*(*X*) is complete. Suppose that the following conditions are satisfied:

- *d*(*fx*, *fy*)⪯*λ*[*d*(*gx*, *fy*) + *d*(*gy*, *fy*)] for all *x*, *y* ∈ *X* with *gx*⊑*gy*, where *λ* ∈ [0,1/2);
- *f* is *g*-nondecreasing;
- there exists *x*_0_ ∈ *X* such that *gx*_0_⊑*fx*_0_;
- if {*gx*_*n*_} were any nondecreasing sequence in *X* converging to some *gz*, then *gx*_*n*_⊑*gz* for all *n* and *gz*⊑*g* *gz*.

Then, *f* and *g* have a coincidence point. Furthermore, if *f* and *g* are weakly compatible then they have a common fixed point. In addition, the set of common fixed points of *f* and *g* is *g*-well ordered if and only if the common fixed point of *f* and *g* is unique.

The following example shows that the results of this paper are a proper generalization of the results of Malhotra et al. [26] and Vetro [14].

**Example 15**

Let *X* = {1,2, 3,4} and *E* = *C*_ℝ_^1^[0,1] with ||*x*|| = ||*x*||_*∞*_ + ||*x*′||_*∞*_, *P* = {*x*(*t*) : *x*(*t*) ≥ 0 for *t* ∈ [0,1]}. Define *d* : *X* × *X* → *E* as follows:

(29) d(1,2)=d(2,1)=3et,d(2,3)=d(3,2)=d(1,3)=d(3,1)=et,d(1,4)=d(4,1)=d(2,4)=d(4,2)=d(3,4)=d(4,3)=4et,d(x,y)=θ if  x=y.

Then, (*X*, *d*) is a complete nonnormal cone rectangular metric space but not cone metric space. Define mappings *f*,  *g* : *X* → *X* and partial order “⊑” on *X* as follows:

(30) $$\begin{array}{c} f=\left(\begin{array}{cccc} 1 & 2 & 3 & 4\\ 1 & 3 & 3 & 1 \end{array}\right),\\ g=\left(\begin{array}{cccc} 1 & 2 & 3 & 4\\ 1 & 2 & 2 & 3 \end{array}\right),\\ ⊑=\left\{\left(1,1\right),\left(2,2\right),\left(3,3\right),\left(4,4\right),\left(1,2\right),\left(1,3\right)\right\}. \end{array}$$

Then it is easy to verify that *f* is an ordered *g*-weak contraction in (*X*, ⊑, *d*) with *λ* ∈ [1/3,1), *μ* = *δ* = 0. Indeed, we have to check the validity of (6) only for (*x*, *y*) = (1,2), (1,4). Then,

(31) $$\begin{array}{c} d\left(f1,f2\right)=d\left(1,3\right)=e^{t},\\ \lambda d\left(g1,g2\right)+\mu d\left(g1,f1\right)+\delta d\left(g2,f2\right)=e^{t}\left[3\lambda +\delta \right]; \end{array}$$

therefore, (6) holds for *λ* ∈ [1/3,1), *μ* = *δ* = 0. Again,

(32) $$\begin{array}{c} d\left(f1,f4\right)=d\left(1,1\right)=\theta ; \end{array}$$

therefore, (6) holds for arbitrary *λ*, *δ*, and *μ* such that *λ* + *μ* + *δ* < 1.

All other conditions of Theorem 10 are satisfied and 1 is the unique common fixed point of *f* and *g*. Note that *f* is not an ordered Reich-type contraction. Indeed, for point (*x*, *y*) = (1,3) there are no *λ*, *μ*, *δ* ∈ [0,1) such that condition (5) is satisfied. Therefore, the results of Malhotra et al. [26] are not applicable here.

The following example illustrates the crucial role of weak compatibility of mappings for the existence of common fixed point in Theorem 10.

**Example 16**

Let (*X*, *d*) be the cone rectangular metric space as in Example 15. Then, (*X*, *d*) is a complete nonnormal cone rectangular metric space but not cone metric space. Define mappings *f*, *g* : *X* → *X* and partial order “⊑” on *X* as follows:

(33) $$\begin{array}{c} f=\left(\begin{array}{cccc} 1 & 2 & 3 & 4\\ 2 & 2 & 2 & 3 \end{array}\right),\\ g=\left(\begin{array}{cccc} 1 & 2 & 3 & 4\\ 1 & 3 & 2 & 4 \end{array}\right),\\ ⊑=\left\{\left(1,1\right),\left(2,2\right),\left(3,3\right),\left(4,4\right),\left(2,4\right),\left(2,3\right),\left(1,3\right)\right\}. \end{array}$$

Then, it is easy to verify that *f* is an ordered *g*-weak contraction in (*X*, ⊑, *d*) with *λ* = *δ* ∈ [1/8,1/2),  *μ* = 0. Indeed, we have to check the validity of (6) only for (*x*, *y*) = (3,4), (3,2), (1,2). Then,

(34) $$\begin{array}{c} d\left(f3,f4\right)=d\left(2,3\right)=e^{t},\\ \lambda d\left(g3,g4\right)+\mu d\left(g3,f3\right)+\delta d\left(g4,f4\right)=4e^{t}\left[\lambda +\delta \right]; \end{array}$$

Therefore, (6) holds for *λ* = *δ* ∈ [1/8,1/2), *μ* = 0. Again,

(35) $$\begin{array}{c} d\left(f3,f2\right)=d\left(2,2\right)=\theta ; \end{array}$$

Therefore, (6) holds for arbitrary *λ*, *δ*, and *μ* such that *λ* + *μ* + *δ* < 1.

Similarly, for (*x*, *y*) = (1,2), and condition (6) holds for arbitrary *λ*, *δ*, and *μ* such that *λ* + *μ* + *δ* < 1.

All other conditions of Theorem 10 (except *f* and *g* are weakly compatible) are satisfied and 3 is a coincidence point of *f* and *g*. Note that *f*3 = *g*3 = 2; that is,  3 is a coincidence point of *f* and *g* but *fg*3 ≠ *gf*3; therefore, *f* and *g* are not weakly compatible and have no common fixed point.

In the following theorem, the conditions on *f*, “nondecreasing” and “completeness of space,” are replaced by another condition.

**Theorem 17**

Let (*X*, ⊑, *d*) be an ordered cone rectangular metric space and *f*, *g* : *X* → *X* is two mappings such that *f*(*X*) ⊂ *g*(*X*). Suppose that the following conditions are satisfied:

- *f* is an ordered *g*-weak contraction that satisfies (6);
- there exists *u* ∈ *X* such that *gu*⊑*fu* and *d*(*gu*, *fu*)⪯*d*(*gx*, *fx*) for all *x* ∈ *X*.

Then, *f* and *g* have a coincidence point. Furthermore, if *f* and *g* are weakly compatible, then they have a common fixed point. In addition, the set of common fixed points of *f* and *g* is *g*-well ordered if and only if the common fixed point of *f* and *g* is unique.

**Proof**

Let *F*(*x*) = *d*(*gx*, *fx*) for all *x* ∈ *X* and *gz* = *fu* for some *z* ∈ *X* (which is possible, since *f*(*X*) ⊂ *g*(*X*)); then *F*(*u*)⪯*F*(*x*) for all *x* ∈ *X*. If *F*(*u*) = *θ*, then *gu* = *fu*; that is,  *u* is a coincidence point of *f* and *g*. If *θ*≺*F*(*u*), then by assumption (B) *gu*⊑*fu*, so *gu*⊑*gz*, and by (A), we obtain

(36) F(z)=d(gz,fz)=d(fu,fz)⪯λd(gu,gz)+μd(gu,fu)+δd(gz,fz)=λd(gu,fu)+μd(gu,fu)+δd(gz,fz)=λF(u)+μF(u)+δF(z),F(z)⪯λ+μ1−δF(u)≺F(u)        (as  λ+μ+δ<1),

a contradiction. Therefore, we must have *F*(*u*) = *θ*; that is,  *gu* = *fu*, and so *u* is a coincidence point of *f* and *g*.

The existence, necessary and sufficient condition for uniqueness of common fixed point follows from a similar process as used in Theorem 10.
